# Medicinal Plants of Ohio

**Published:** 1849-12

**Authors:** John M. Bigelow


*Medicinal Plants of Ohio.* By JOHN M. BIGELOW, M. D.—The November number of the Ohio Medical and Surgical Journal contains a list of the medicinal plants of Ohio, not embraced in Wood & Bache's U. S. Dispensatory, together with a brief account of their properties, by John M. Bigelow, M. D. The list is extensive, and, as it seems to us, Dr. B. has rendered a valuable service to American Medical Botany, by this publication of the fruits of his labors in that interesting and important depatment of medical knowledge. We learn from the paper referred to, which has also been issued in a pamphlet form by J. H. Riley & Co., of Columbus, Ohio, that the author is chairman of a committee appointed by the Ohio State Medical Society, to report on the "Materia Medica and Botany of Ohio," at its annual meeting in June, 1850.

